# Supra­molecular assembly of mebendazolium and di­hydrogen phosphate ions in a new anthelmintic salt

**DOI:** 10.1107/S2056989025000714

**Published:** 2025-02-04

**Authors:** Eduardo L. Gutiérrez, Marcos G. Russo, Griselda E. Narda, Elena V. Brusau, Alejandro P. Ayala, Javier Ellena

**Affiliations:** a Instituto de Investigaciones en Tecnología Química (INTEQUI CONICET-UNSL) Área de Química Orgánica Facultad de Química Bioquímica y Farmacia Universidad Nacional de San Luis (UNSL) D5700APC San Luis Argentina; b Departamento de Física Universidade Federal do Ceará (UFC) 60440-900 Fortaleza CE Brazil; c Instituto de Física de São Carlos (IFSC) Universidade de São Paulo (USP) 13566-590 São Carlos SP Brazil; Universidad Nacional Autónoma de México, México

**Keywords:** pharmaceutical materials, mebendazole, supra­molecular synthon, single-crystal X-ray diffraction, Hirshfeld surface analysis

## Abstract

A new mebendazolium di­hydrogen phosphate phospho­ric acid solid material was obtained and characterized by single-crystal X-ray diffraction and complementary solid-state techniques {systematic name: 5-benzoyl-2-[(meth­oxy­carbon­yl)amino]-1*H*-1,3-benzo­diazol-3-ium di­hydrogen phosphate–phos­pho­ric acid (1/1), C_16_H_14_N_3_O_3_^+^·H_2_PO_4_^−^·H_3_PO_4_}. Structure solution confirmed proton transfer from phospho­ric acid towards the basic imidazole ring of mebendazole. The mebendazolium cation and the di­hydrogen phosphate anion assemble in the solid state in a cyclic hydrogen-bond-driven supra­molecular motif, as observed in all mebendazolium/oxyanions structures reported in the literature. This salt crystallizes in the monoclinic *P*2_1_/*c* (No. 14) space group. A detailed study of the crystal structure performed by atom-to-atom and global Hirshfeld surface analysis indicates that several hydrogen bonds act as the main inter­molecular inter­actions stabillizing the material. The new material is stable up to 458 K.

## Abstract 2

A new mebendazolium di­hydrogen phosphate phospho­ric acid solid compound was obtained. As expected, the mebendazolium cation and the di­hydrogen phosphate anion assemble in the solid state in an *R*_2_^2^(8) hydrogen-bond-driven supra­molecular motif.

## 1. Chemical context

Mebendazole [MBZ, methyl *N*-(5-benzoyl­benzimidazol-2-yl) carbamate, in red in the scheme) is a synthetic broad-spectrum benzimidazole-derivative anthelmintic API (Active Pharmaceutical Ingredient) (Martins *et al.*, 2009 ▸). MBZ is used extensively in human medicine being administrated orally as tablet formulation or suspension and is included in the World Health Organization (WHO) Model List of Essential Drugs (Agatonovic-Kustrin *et al.*, 2008 ▸).

MBZ can exist as several tautomers, leading to the existence of three solid forms (*i.e*., desmotropes) with remarkable differences in their physicochemical properties and bioavailability (Ayala *et al.*, 2008 ▸). MBZ desmotropes can undergo tautomeric inter­conversion in the solid state under the effect of heat and moisture. In particular, investigations have indicated that the pharmaceutically preferred form C evolves into the inactive form A in commercial formulations (Calvo *et al.*, 2016 ▸). However, our results indicate that salification of the API prevents tautomeric transformation (Gutiérrez *et al.*, 2020 ▸).

Our objective is to design, on the basis of supra­molecular assembly, new MBZ multicomponent systems aiming to obtain materials incorporating the API and avoiding desmotrope inter­conversion and exhibiting a solubility and dissolution profile similar to those of the MBZ therapeutically preferred form C. In previous works, we reported the high statistical probability of formation of an 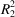
(8) supra­molecular heterosynthon (Bernstein *et al.*, 1995 ▸; Motherwell *et al.*, 2000 ▸) between protonated mebendazole mol­ecules (*i.e*., the mebendazolium cation, shown in red in the scheme) and polyatomic oxyanions (Gutiérrez *et al.*, 2018 ▸, 2024 ▸).
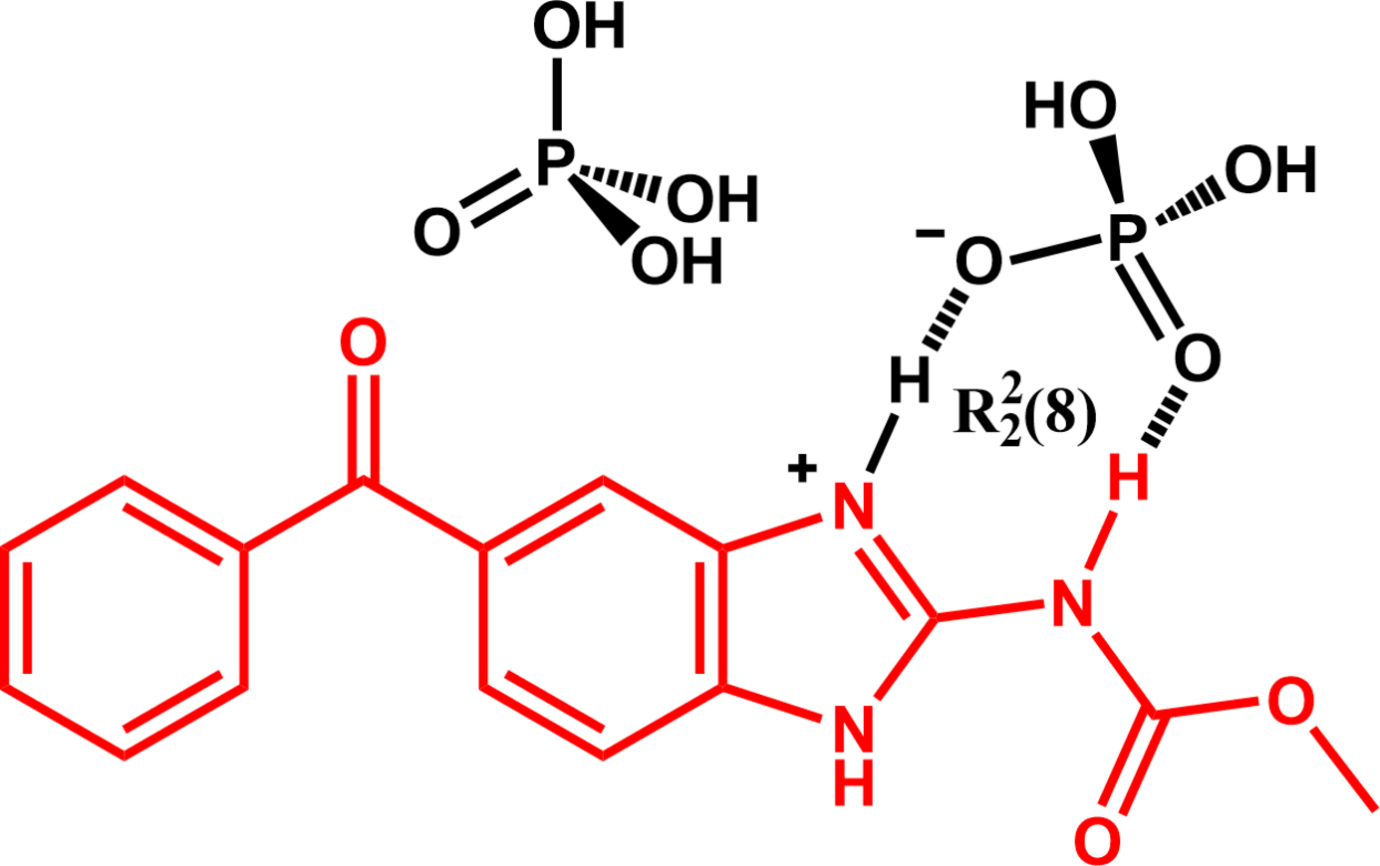


In the context of screening for new mebendazole multicomponent materials, we obtained a new mebendazolium di­hydrogen phosphate phospho­ric acid compound. This material can be considered as an ionic cocrystal (*i.e*., a cocrystal of a salt), since the phospho­ric acid mol­ecules are connected with the ionic components of the salt through non-covalent inter­actions (*i.e*., hydrogen-bonds) (Pavlović *et al.*, 2024 ▸; Smith *et al.*, 2013 ▸). Phospho­ric acid and its anions are approved coformers by the Food and Drug Administration (FDA) and U·S. Department of Health and Human Services (FDA, 2024 ▸), which could make this new material suitable for pharmaceutical formulations.

## 2. Structural commentary

**Figure 1:**
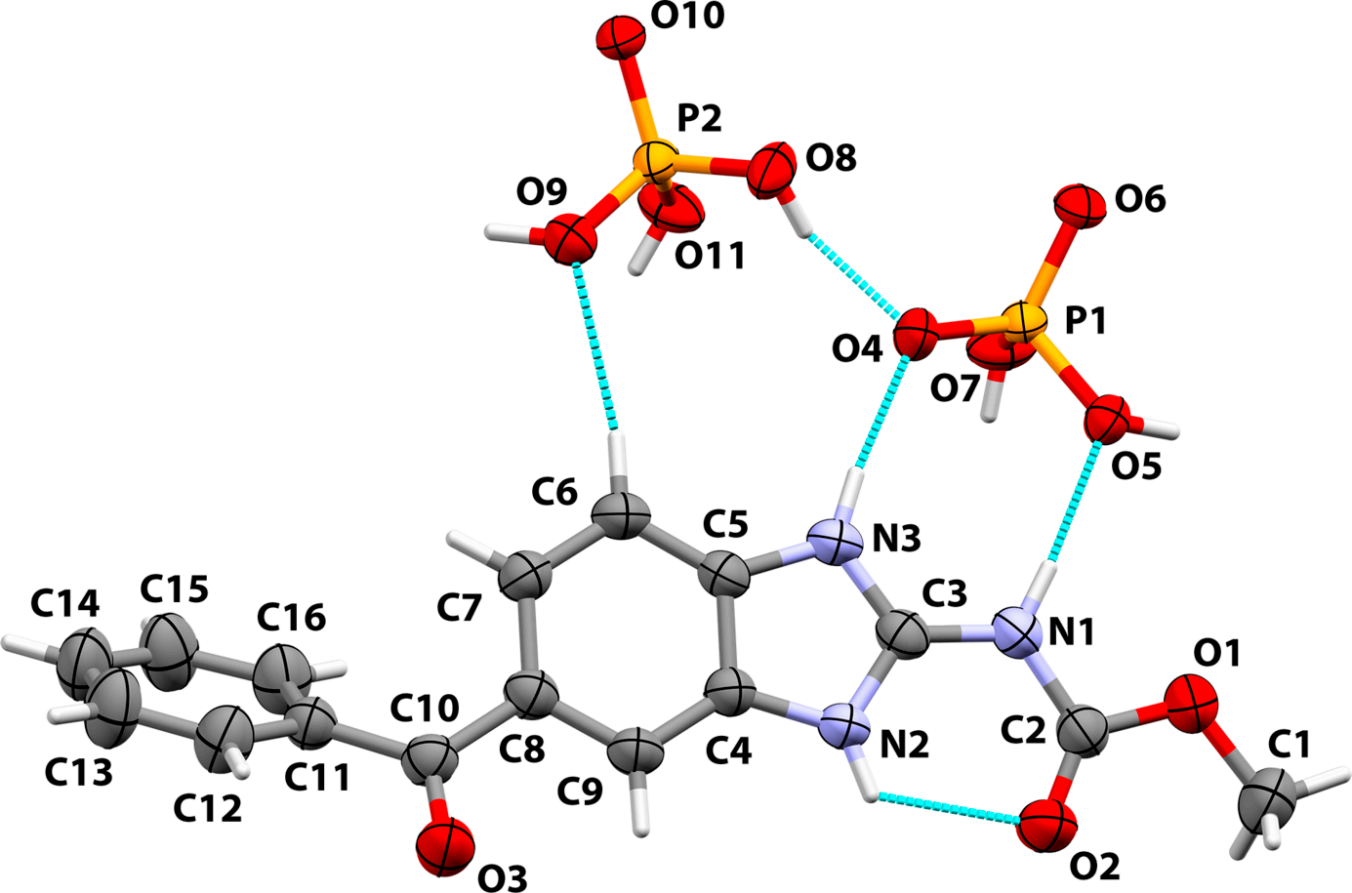
View of the asymmetric unit in the crystal of MBZH·PO_2_(OH)_2_·PO(OH)_3_, showing the atom labeling and the 50% probability ellipsoids for non-hydrogen atoms. The hydrogen atoms are shown as sticks of arbitrary radii (color code. C: gray; H: white; O: red; N: blue; P: orange).

The solution of the structure confirmed the crystallization of a new mebendazole material, which is a mebendazolium (MBZH^+^) di­hydrogen phosphate phospho­ric acid compound, MBZH·PO_2_(OH)_2_·PO(OH)_3_, with 1:1:1 stoichiometry. Δp*K_a_* between the MBZH^+^ cation and phospho­ric acid is 1.31 (see supporting information). This value falls in the ‘grey area’ for predictions on the location of the acidic proton. Around Δp*K*_a_ ∼1, predicting the location of the acid proton based on aqueous Δp*K_a_* data alone is not possible: the model predicts a very similar likelihood of observing salts or cocrystals (Cruz-Cabeza, 2012 ▸). This salt crystallizes in the monoclinic *P*2_1_/*c* (No. 14) space group. An *ORTEP*-type view of the asymmetric unit of MBZH·PO_2_(OH)_2_·PO(OH)_3_ is shown in Fig. 1 ▸. The atom labeling is according to the nomenclature used in previous related works.

In the Supporting Information file we present a detailed analysis of the bond-length changes in the imidazole ring and the carbamate moiety, which confirms the protonation of the API. The MBZH^+^ cation is found in a conformation in which the carbamate moiety is coplanar with the benzimidazole ring. This conformation is favored by (i) the conjugation of these moieties and the partial double-bond character of the bonds O2—C2, C2—N1 and N1—C3 reinforced by the positive charge of the mol­ecule, and (ii) the intra­molecular resonance-assisted hydrogen bond between N2 and O2 [H⋯*A*: 2.10 (3) Å, 122 (3)°]. The benzimidazole and the benzene rings are not coplanar: the dihedral angle between the least-squares planes passing through the benzene (root-mean-square deviation, r.m.s.d., of fitted atoms: 0.004 Å) and the benzimidazole (r.m.s.d.: 0.003 Å) rings is 54.50 (17)°.

## 3. Supra­molecular features

**Figure 2:**
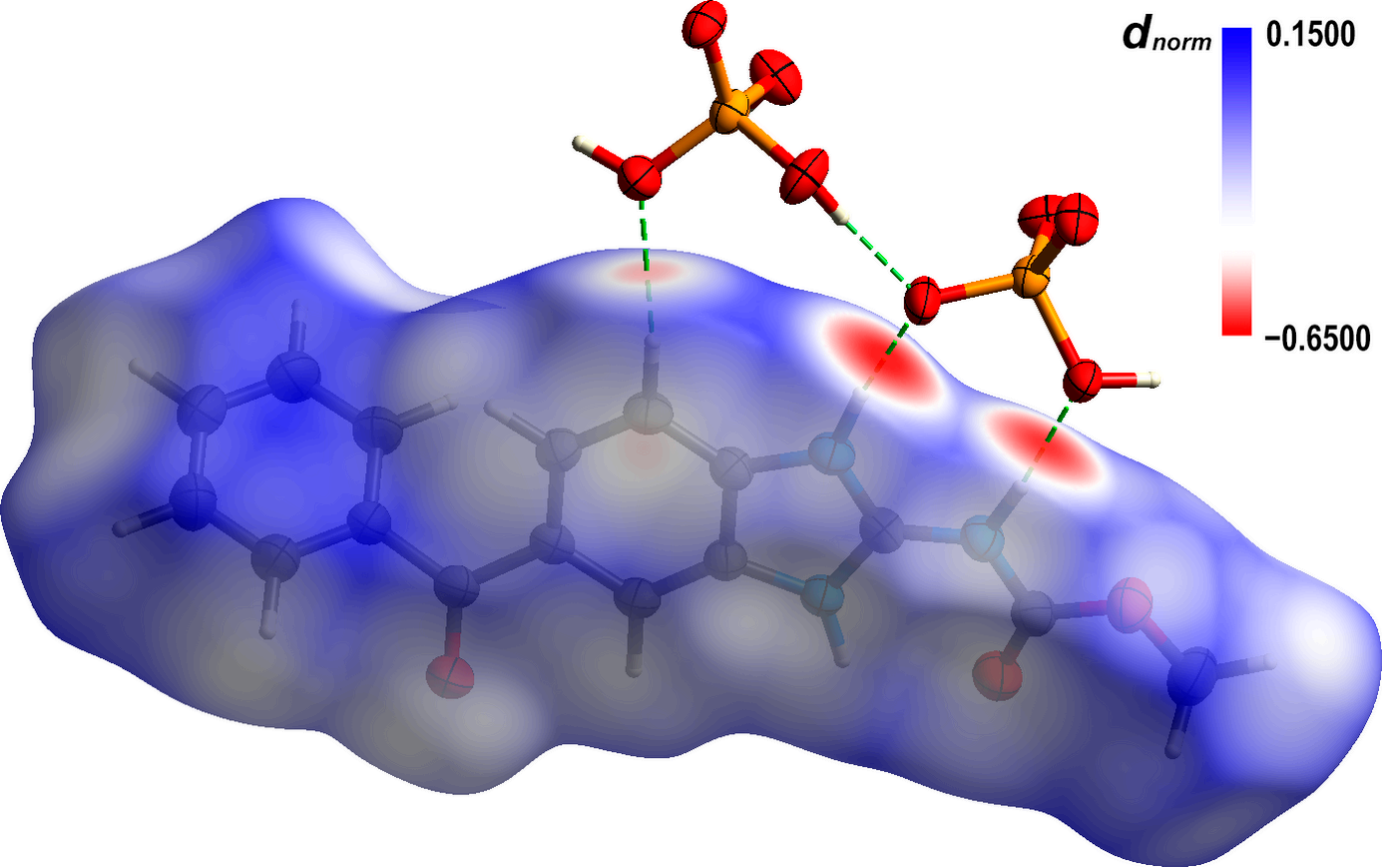
Mebendazolium cation mapped with the *d_norm_* function on the Hirshfeld surface (color code. C: gray; H: white; O: red; N: blue; P: orange).

Hirshfeld surface (HS) analysis and energy calculations (HF/3-21G model) were performed using the software *Crystal Explorer 21.5* (Mackenzie *et al.*, 2017 ▸; Spackman *et al.*, 2021 ▸) with the CIF as the input file. The Hirshfeld surface for the title compound was mapped with the *d_norm_* function over the range −0.025 to 0.750 a.u. (color code: from blue – distances longer than sum of van der Waals radii – through white to red – distances shorter than sum of van der Waals radii). Through HS analysis, it was confirmed that main inter­molecular inter­actions stabilizing the crystal structure are several hydrogen bonds. Fig. 2 ▸ shows the HS of the MBZH^+^ cation mapped with the *d_norm_* function (Spackman *et al.*, 2009 ▸), where the red spots are the regions in which the inter­atomic distances are shorter than the sum of the van der Waals radii and correspond to the hydrogen bonds.

**Figure 3:**
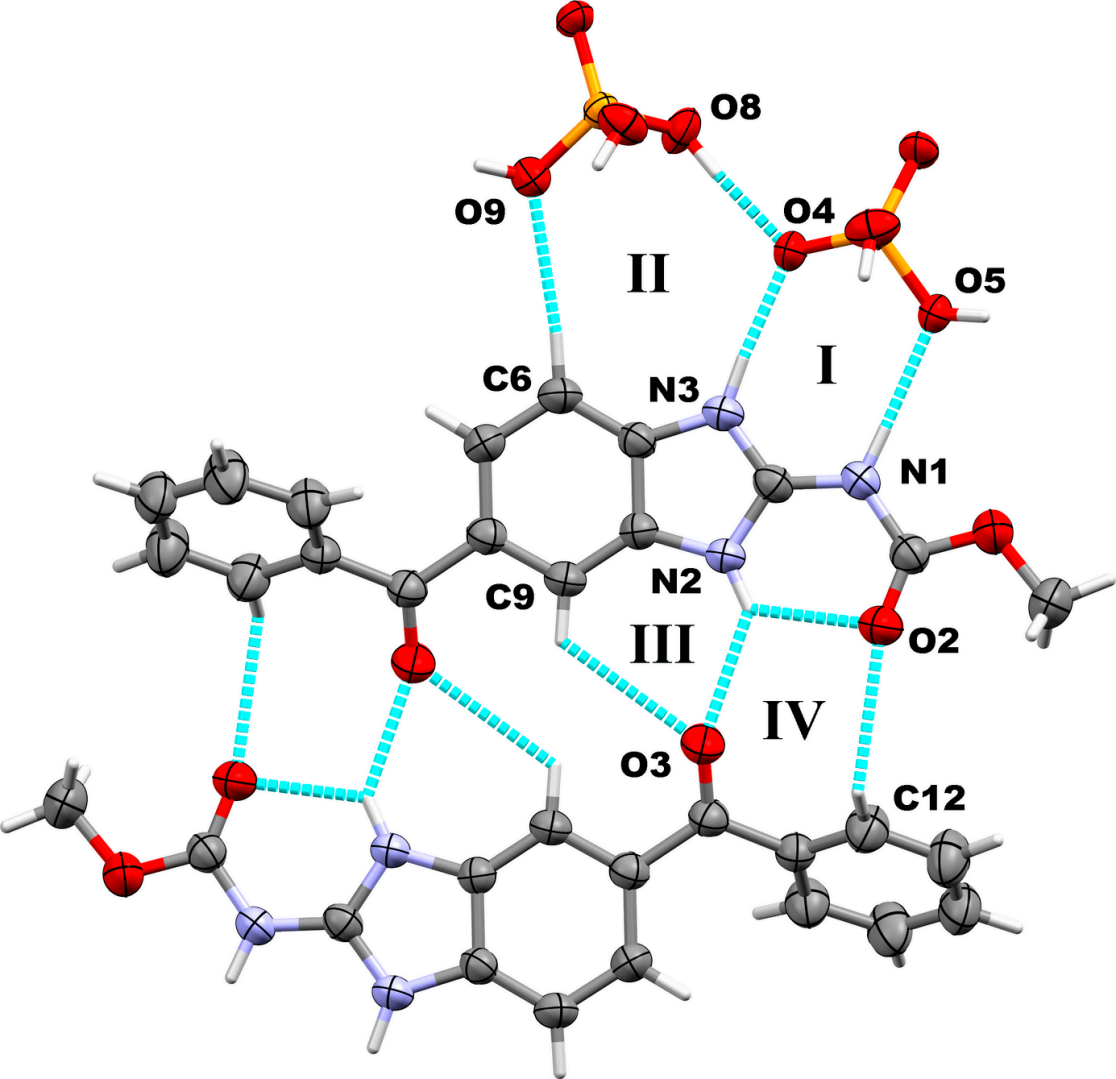
Hydrogen-bonding patterns found in the crystal packing of MBZH·PO_2_(OH)_2_·PO(OH)_3_ (color code. C: gray; H: white; O: red; N: blue; P: orange).

The MBZH^+^ cations and PO_2_(OH)_2_^−^ anions form a heterosynthon described by the 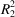
(8) graph-set motif (Bernstein *et al.*, 1995 ▸; Motherwell *et al.*, 2000 ▸) (scheme and Fig. 3 ▸, labeled as **I**), which is stabilized by moderate hydrogen bonds (Steiner, 2002 ▸). N3 and N1 act as proton donors, while O4 and O5 are acceptors, establishing two hydrogen-bond schemes: N3—H3⋯O4 [1.86 (2) Å] and N1—H1⋯O5 [1.96 (2) Å]. This supra­molecular motif is not planar according to the dihedral angle of 29.1 (4)° between the planes defined by the atoms N3, C3, and N1, and O4, P1, and O5, respectively. This inter­action is inherently stabilizing, with a calculated value of −477.2 kJ mol^−1^. The second most stabilizing inter­action according to our calculations is a predominantly electrostatic inter­action between the MBZH^+^ cation and a second PO_2_(OH)_2_^−^ anion along the *b-*axis direction, which accounts for −393.5 kJ mol^−1^ (Fig. S1*A*). Three other distinct supra­molecular motifs are observed in the crystal packing, involving more complex hydrogen-bonding patterns. On the one hand, a phospho­ric acid mol­ecule is involved in a cyclic arrangement adjacent to the motif previously described. This motif is labeled as **II** in Fig. 3 ▸ and is described by the 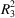
(10) graph-set motif. The inter­action between the phospho­ric acid mol­ecule and the MBZH^+^ cation is predominantly electrostatic and destabilizing (127.3 kJ mol^−1^). On the other hand, two MBZH^+^ cations are assembled by the two cyclic motifs labeled **III** [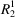
(6)] and **IV** [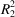
(8)] in Fig. 3 ▸, bringing together a pair of inversion-related cations. This arrangement is overall destabilizing (33.4 kJ mol^−1^). Finally, we also found C—H⋯π (114.1 kJ mol^−1^) and carbon­yl⋯π inter­actions relating adjacent MBZH^+^ cations (Fig. S1*B*), which are overall destabilizing. Hydrogen-bonding geometry parameters are shown in Table 1 ▸.

## 4. Database survey

A survey for the structure of the MBZH^+^ cation in the Cambridge Structural Database [CSD version: 5.46 (November 2024); Groom *et al.*, 2016 ▸], using *ConQuest 5.45* software (Bruno *et al.*, 2002 ▸), gave eleven matches, eight of them being anhydrous salts of oxyanions or carboxyl­ates. All eight structures, regardless of the space group of the compound and the geometry of the anions, feature the same 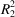
(8) supra­molecular motif that was found in the title compound between the MBZH^+^ cation and the respective anion. Table S1 summarizes the relevant features of the hydrogen-bonding patterns related to the 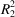
(8) supra­molecular motif found in the reported mebendazolium salts (Fig. S2) for further comparison with the compound reported here.

## 5. Synthesis and crystallization

A 15 mg (0.05 mmol) sample of MBZ desmotrope C was suspended in 30 mL of methanol at room temperature with constant magnetic stirring (1000 r.p.m.). An excess of phospho­ric acid (85% *w*/*w*, purchased from UCB) was added (3 mL) and the suspension was heated up to 333 K until complete dissolution of the solid. The resulting solution was filtered and covered with a Parafilm foil with small holes to regulate the speed of the evaporation of the solvent and was left to evaporate at room temperature. After approximately twenty days, the formation of small, colorless prismatic crystals was observed. These crystals were separated by filtration and washed several times with distilled water and then with *n*-hexane. The crystalline material was then stored at room temperature for further characterization (Fig. S3).

FTIR spectroscopy and powder X-ray diffraction (PXRD) were used to check the identity and purity of the bulk material. Both techniques confirmed the obtention of a new material. MBZ, di­hydrogen phosphate and phospho­ric acid characteristic vibrational modes were observed in the FTIR spectrum (Fig. S4) of the solid. In particular, the carbamate C=O stretching mode of MBZ, which is very sensitive to the crystalline environment (Ayala *et al.*, 2008 ▸), was observed at 1753 cm^−1^. This band is extensively used to identify and quantify the MBZ desmotropes even in pharmaceutical products (Calvo *et al.*, 2016 ▸) and is observed at 1730 cm^−1^, 1697 cm^−1^ and 1715 cm^−1^ in MBZ A (Ferreira *et al.*, 2010 ▸), B (Bravetti *et al.*, 2022 ▸) and C (Martins *et al.*, 2009 ▸) spectra, respectively. The inorganic acid and the anion contribute to the broadening of the band in the range 3000–2500 cm^−1^, these species also being responsible for the band centered at 2350 cm^−1^. While several vibrational modes of the inorganic moieties are overlapped with those derived from mebendazolium, the ν(P—OH) vibration modes clearly appear as several bands in the 1200–900 cm^−1^ zone. The ν_as_ (O—P—O) vibration mode can be assigned to the bands at 553 and 496 cm^−1^. The assignment of selected vibrational modes on the FTIR spectrum is shown in Table S2.

The PXRD pattern (Fig. S5) of the bulk material was compared with the calculated patterns of MBZ A, B, and C, and with that calculated for the refined structure. Calculated patterns were obtained using *Mercury* (Macrae *et al.*, 2020 ▸) with the CIFs as input. This comparison confirmed both the identity and the purity of the the new material as its pattern does not match with those of MBZ desmotropes and no characteristic peaks other than those of the new solid are present in the experimental pattern. In Table S3, we present a list of the main reflexions in the experimental PXRD pattern.

The material is stable up to 458 K, when the endothermic elimination of phospho­ric acid takes place (experimental mass loss, exp .: 15.90%, theoretical mass loss, theor.: 19.95%). The degradation of MBZH^+^ mol­ecule starts at 634 K and involves the elimination of the (meth­yl)formyl moiety (overall exp .: 36.91%, theor.: 31.69%). The differences of approx. 4% between the experimental and theoretical values are attributed to solvent and/or phospho­ric acid residues in the sample. Thermogravimetric Analysis and Differential Scanning Calorimetry curves are shown in Fig. S6.

## 6. Refinement

Crystal data, data collection and structure refinement details are summarized in Table 2 ▸. All hydrogen atoms were refined using a DFIX restraint to ensure chemically reasonable bond lengths and angles, with their *U*_iso_(H) values constrained to 1.5 times the *U_eq_* of their pivot atoms for terminal *sp*^3^ carbon atoms and 1.2 times for all other carbon atoms. The structure was solved as a pseudomerohedral two-component twin [0.8510 (9)/0.1490 (9)] arising from a twofold axis using the twin law (−1 0 0, 0 − 1 0, 1 0 1).

## Supplementary Material

Crystal structure: contains datablock(s) I. DOI: 10.1107/S2056989025000714/jq2039sup1.cif

Structure factors: contains datablock(s) I. DOI: 10.1107/S2056989025000714/jq2039Isup2.hkl

Supporting information file. DOI: 10.1107/S2056989025000714/jq2039sup4.pdf

Supporting information file. DOI: 10.1107/S2056989025000714/jq2039Isup4.cml

CCDC reference: 2381432

Additional supporting information:  crystallographic information; 3D view; checkCIF report

## Figures and Tables

**Table 1:** Hydrogen-bond geometry (Å, °)

*D*—H⋯*A*	*D*—H	H⋯*A*	*D*⋯*A*	*D*—H⋯*A*
O5—H5⋯O10^i^	0.84 (2)	1.74 (2)	2.578 (3)	175 (4)
O9—H9*A*⋯O6^ii^	0.85 (2)	1.72 (2)	2.568 (3)	177 (5)
O8—H8⋯O4	0.87 (2)	1.68 (2)	2.544 (3)	171 (4)
O11—H11⋯O10^iii^	0.84 (2)	1.79 (2)	2.629 (3)	178 (4)
O7—H7*A*⋯O6^iii^	0.85 (2)	1.79 (2)	2.594 (3)	157 (4)
N2—H2⋯O2	0.90 (2)	2.10 (3)	2.680 (4)	122 (3)
N3—H3⋯O4^iv^	0.90 (2)	1.82 (2)	2.717 (3)	177 (3)
N1—H1⋯O5^iv^	0.88 (2)	1.96 (2)	2.831 (3)	174 (3)

Symmetry codes: (i) 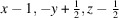; (ii) 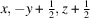; (iii) 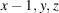; (iv) 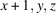.

**Table 2:** Experimental details

Crystal data	
Chemical formula	C_16_H_14_N_3_O_3_^+^·H_2_O_4_P^−^·H_3_O_4_P
*M*_r_	491.28
Crystal system, space group	Monoclinic, *P*2_1_/*c*
Temperature (K)	302
*a*, *b*, *c* (Å)	4.7217 (3), 26.167 (2), 16.7888 (12)
β (°)	98.082 (5)
*V* (Å^3^)	2053.7 (3)
*Z*	4
Radiation type	Cu *K*α
μ (mm^−1^)	2.55
Crystal size (mm)	0.18 × 0.07 × 0.03

Data collection	
Diffractometer	Bruker D8 VENTURE dual wavelength Mo/Cu
Absorption correction	Multi-scan (*SADABS*; Krause *et al.*, 2015 ▸)
*T*_min_, *T*_max_	0.647, 0.754
No. of measured, independent and observed [*I* > 2σ(*I*)] reflections	30433, 4094, 3363
*R*_int_	0.076
(sin θ/λ)_max_ (Å^−1^)	0.618

Refinement	
*R*[*F*^2^ > 2σ(*F*^2^)], *wR*(*F*^2^), *S*	0.044, 0.105, 1.06
No. of reflections	4094
No. of parameters	348
No. of restraints	19
H-atom treatment	Only H-atom coordinates refined
Δρ_max_, Δρ_min_ (e Å^−3^)	0.29, −0.32

Computer programs: *APEX4* (Bruker, 2021 ▸), *SAINT* (Bruker, 2018 ▸), *SHELXT* (Sheldrick, 2015*a* ▸), *SHELXL* (Sheldrick, 2015*b* ▸) and *OLEX2* (Dolomanov *et al.*, 2009 ▸).

## supplementary crystallographic information

### 5-Benzoyl-2-[(methoxycarbonyl)amino]-1H-1,3-benzodiazol-3-ium dihydrogen phosphate–phosphoric acid (1/1) Crystal data

**Table:**

C_16_H_14_N_3_O_3_^+^·H_2_O_4_P^−^·H_3_O_4_P	*F*(000) = 1016
*M**_r_* = 491.28	*D*_x_ = 1.589 Mg m^−^^3^
Monoclinic, *P*2_1_/*c*	Cu *K*α radiation, λ = 1.54178 Å
*a* = 4.7217 (3) Å	Cell parameters from 9651 reflections
*b* = 26.167 (2) Å	θ = 3.2–72.3°
*c* = 16.7888 (12) Å	µ = 2.55 mm^−^^1^
β = 98.082 (5)°	*T* = 302 K
*V* = 2053.7 (3) Å^3^	Prism, clear light colourless
*Z* = 4	0.18 × 0.07 × 0.03 mm

### 5-Benzoyl-2-[(methoxycarbonyl)amino]-1H-1,3-benzodiazol-3-ium dihydrogen phosphate–phosphoric acid (1/1) Data collection

**Table:**

Bruker D8 VENTURE dual wavelength Mo/Cu diffractometer	4094 independent reflections
Radiation source: microfocus sealed X-ray tube, Incoatec Iµs HB	3363 reflections with *I* > 2σ(*I*)
Multilayer mirrors monochromator	*R*_int_ = 0.076
Detector resolution: 7.39 pixels mm^-1^	θ_max_ = 72.4°, θ_min_ = 2.7°
ω and φ scans	*h* = −5→5
Absorption correction: multi-scan (SADABS; Krause *et al.*, 2015)	*k* = −32→31
*T*_min_ = 0.647, *T*_max_ = 0.754	*l* = −20→20
30433 measured reflections	

### 5-Benzoyl-2-[(methoxycarbonyl)amino]-1H-1,3-benzodiazol-3-ium dihydrogen phosphate–phosphoric acid (1/1) Refinement

**Table:**

Refinement on *F*^2^	Hydrogen site location: difference Fourier map
Least-squares matrix: full	Only H-atom coordinates refined
*R*[*F*^2^ > 2σ(*F*^2^)] = 0.044	*w* = 1/[σ^2^(*F*_o_^2^) + (0.0387*P*)^2^ + 1.4065*P*] where *P* = (*F*_o_^2^ + 2*F*_c_^2^)/3
*wR*(*F*^2^) = 0.105	(Δ/σ)_max_ < 0.001
*S* = 1.06	Δρ_max_ = 0.29 e Å^−^^3^
4094 reflections	Δρ_min_ = −0.32 e Å^−^^3^
348 parameters	Extinction correction: SHELXL-2019/1 (Sheldrick 2015b), Fc^*^=kFc[1+0.001xFc^2^λ^3^/sin(2θ)]^-1/4^
19 restraints	Extinction coefficient: 0.00153 (15)
Primary atom site location: dual	

### 5-Benzoyl-2-[(methoxycarbonyl)amino]-1H-1,3-benzodiazol-3-ium dihydrogen phosphate–phosphoric acid (1/1) Special details

**Table:**

Geometry. All esds (except the esd in the dihedral angle between two l.s. planes) are estimated using the full covariance matrix. The cell esds are taken into account individually in the estimation of esds in distances, angles and torsion angles; correlations between esds in cell parameters are only used when they are defined by crystal symmetry. An approximate (isotropic) treatment of cell esds is used for estimating esds involving l.s. planes.
Refinement. Refined as a 2-component twin. Single crystal X-ray diffraction data (φ scans and ω scans with κ and θ offsets) were collected on a Bruker D8 Venture κ-geometry diffractometer equipped with a Photon II CPAD detector and a IµS 3.0 Incoatec Cu Kα (λ = 1.54178 Å) microfocus source. A suitable crystal for the compound was chosen and mounted on a Kapton fiber with a MiTeGen MicroMount using immersion oil. The APEX 4 software was used for the unit cell determination and data collection (Bruker AXS Inc, 2021). The data reduction and global cell refinement were made using the Bruker SAINT+ software package (Bruker AXS Inc, 2019), and a multi-scan absorption correction was performed with SADABS (Krause *et al.*, 2015). Using the Olex2 (Dolomanov *et al.*, 2009) interface program to the SHELX suite, the structure was solved by the intrinsic phasing method implemented in ShelXT (Sheldrick, 2015b), allowing the location of most of the non-hydrogen atoms. The remaining non-hydrogen atoms were located from difference Fourier maps calculated from successive full-matrix least-squares refinement cycles on F^2^ with ShelXL (Sheldrick, 2015a) and refined using anisotropic displacement parameters.

### 5-Benzoyl-2-[(methoxycarbonyl)amino]-1H-1,3-benzodiazol-3-ium dihydrogen phosphate–phosphoric acid (1/1) Fractional atomic coordinates and isotropic or equivalent isotropic displacement parameters (Å^2^)

**Table:**

	*x*	*y*	*z*	*U*_iso_*/*U*_eq_	
P2	0.43419 (16)	0.27652 (3)	0.23599 (4)	0.03209 (18)	
P1	0.81274 (16)	0.23835 (3)	0.50120 (4)	0.03376 (19)	
O5	0.2739 (5)	0.31777 (8)	0.17736 (11)	0.0365 (5)	
H5	0.205 (8)	0.3072 (13)	0.1312 (14)	0.055*	
O6	0.6963 (4)	0.25750 (9)	0.20482 (12)	0.0418 (5)	
O4	0.4896 (5)	0.30257 (9)	0.31664 (12)	0.0437 (5)	
O10	1.0793 (4)	0.21012 (9)	0.53275 (12)	0.0411 (5)	
O9	0.7150 (5)	0.27629 (8)	0.56206 (13)	0.0433 (5)	
H9A	0.714 (10)	0.2646 (14)	0.6095 (14)	0.065*	
O8	0.8674 (5)	0.26953 (10)	0.42774 (13)	0.0486 (6)	
H8	0.726 (7)	0.2791 (15)	0.392 (2)	0.073*	
O11	0.5673 (5)	0.20050 (9)	0.47561 (15)	0.0479 (6)	
H11	0.412 (6)	0.2044 (16)	0.494 (2)	0.072*	
O7	0.2289 (5)	0.23040 (9)	0.23605 (16)	0.0494 (6)	
H7A	0.054 (5)	0.2366 (16)	0.239 (3)	0.074*	
O2	0.4932 (6)	0.43652 (10)	0.20414 (14)	0.0556 (6)	
O1	0.7119 (6)	0.39991 (10)	0.10761 (14)	0.0548 (6)	
O3	0.6908 (6)	0.49929 (9)	0.63826 (14)	0.0555 (7)	
N2	0.7646 (6)	0.42602 (10)	0.35453 (15)	0.0386 (6)	
H2	0.617 (6)	0.4437 (12)	0.3285 (19)	0.046*	
N3	1.1169 (6)	0.37031 (10)	0.36730 (16)	0.0408 (6)	
H3	1.242 (7)	0.3475 (11)	0.353 (2)	0.049*	
N1	0.8972 (6)	0.38874 (10)	0.23461 (16)	0.0413 (6)	
H1	1.022 (7)	0.3686 (12)	0.216 (2)	0.050*	
C2	0.6798 (8)	0.41106 (12)	0.18303 (19)	0.0422 (7)	
C3	0.9242 (7)	0.39500 (12)	0.31555 (19)	0.0384 (7)	
C5	1.0829 (7)	0.38557 (12)	0.44514 (18)	0.0375 (7)	
C4	0.8619 (7)	0.42140 (11)	0.43686 (18)	0.0373 (7)	
C9	0.7723 (7)	0.44463 (12)	0.5018 (2)	0.0417 (7)	
H9	0.616 (6)	0.4676 (11)	0.501 (2)	0.050*	
C7	1.1270 (8)	0.39411 (13)	0.5865 (2)	0.0431 (7)	
H7	1.233 (7)	0.3861 (13)	0.6386 (14)	0.052*	
C6	1.2188 (7)	0.37141 (13)	0.5200 (2)	0.0435 (7)	
H6	1.365 (6)	0.3460 (11)	0.528 (2)	0.052*	
C8	0.9056 (7)	0.43107 (12)	0.57789 (19)	0.0400 (7)	
C11	0.8572 (7)	0.43390 (12)	0.72951 (19)	0.0420 (7)	
C10	0.8103 (7)	0.45728 (12)	0.64807 (19)	0.0418 (7)	
C16	0.8251 (10)	0.38162 (13)	0.7409 (2)	0.0563 (10)	
H16	0.761 (9)	0.3601 (13)	0.6951 (17)	0.068*	
C13	0.9560 (12)	0.44489 (16)	0.8727 (2)	0.0694 (12)	
H13	0.994 (10)	0.4680 (14)	0.9179 (19)	0.083*	
C15	0.8548 (12)	0.36190 (15)	0.8181 (2)	0.0670 (12)	
H15	0.821 (10)	0.3261 (8)	0.827 (3)	0.080*	
C12	0.9165 (9)	0.46542 (14)	0.7965 (2)	0.0556 (10)	
H12	0.930 (9)	0.5011 (8)	0.787 (2)	0.067*	
C14	0.9251 (11)	0.39306 (16)	0.8836 (2)	0.0652 (12)	
H14	0.944 (10)	0.3788 (15)	0.9370 (15)	0.078*	
C1	0.4836 (12)	0.4184 (2)	0.0479 (3)	0.0725 (13)	
H1A	0.296 (7)	0.408 (2)	0.061 (3)	0.109*	
H1B	0.523 (12)	0.4067 (19)	−0.0048 (18)	0.109*	
H1C	0.495 (12)	0.4556 (8)	0.054 (3)	0.109*	

### 5-Benzoyl-2-[(methoxycarbonyl)amino]-1H-1,3-benzodiazol-3-ium dihydrogen phosphate–phosphoric acid (1/1) Atomic displacement parameters (Å^2^)

**Table:**

	*U*^11^	*U*^22^	*U*^33^	*U*^12^	*U*^13^	*U*^23^
P2	0.0239 (4)	0.0416 (4)	0.0303 (4)	0.0020 (3)	0.0024 (3)	0.0008 (3)
P1	0.0265 (4)	0.0441 (4)	0.0302 (4)	0.0004 (3)	0.0022 (3)	0.0038 (3)
O5	0.0369 (11)	0.0417 (11)	0.0290 (10)	0.0030 (9)	−0.0014 (9)	−0.0010 (8)
O6	0.0257 (11)	0.0621 (14)	0.0380 (11)	0.0064 (10)	0.0057 (9)	−0.0029 (10)
O4	0.0454 (13)	0.0566 (13)	0.0272 (10)	0.0116 (11)	−0.0019 (9)	−0.0038 (9)
O10	0.0295 (11)	0.0587 (13)	0.0348 (11)	0.0086 (10)	0.0029 (9)	0.0083 (9)
O9	0.0470 (13)	0.0449 (12)	0.0387 (11)	0.0064 (10)	0.0082 (10)	−0.0002 (9)
O8	0.0385 (13)	0.0701 (15)	0.0361 (12)	−0.0018 (12)	0.0013 (9)	0.0203 (11)
O11	0.0290 (12)	0.0548 (14)	0.0613 (15)	−0.0072 (10)	0.0111 (11)	−0.0141 (11)
O7	0.0294 (12)	0.0437 (12)	0.0744 (16)	−0.0031 (10)	0.0047 (11)	0.0111 (11)
O2	0.0539 (16)	0.0607 (15)	0.0509 (14)	0.0188 (13)	0.0026 (12)	−0.0070 (12)
O1	0.0538 (15)	0.0708 (16)	0.0403 (13)	0.0102 (13)	0.0080 (11)	0.0001 (11)
O3	0.0720 (18)	0.0470 (13)	0.0474 (13)	0.0184 (13)	0.0077 (13)	−0.0015 (10)
N2	0.0352 (15)	0.0418 (14)	0.0391 (14)	0.0089 (11)	0.0061 (11)	−0.0013 (11)
N3	0.0354 (15)	0.0442 (15)	0.0434 (15)	0.0087 (12)	0.0077 (12)	−0.0018 (12)
N1	0.0404 (16)	0.0458 (15)	0.0388 (14)	0.0064 (12)	0.0096 (12)	−0.0027 (11)
C2	0.0442 (19)	0.0423 (17)	0.0412 (17)	0.0010 (15)	0.0098 (15)	−0.0005 (13)
C3	0.0347 (17)	0.0396 (16)	0.0419 (17)	−0.0012 (13)	0.0081 (13)	−0.0035 (13)
C5	0.0353 (17)	0.0396 (16)	0.0387 (16)	0.0023 (13)	0.0092 (13)	−0.0013 (12)
C4	0.0368 (17)	0.0372 (15)	0.0376 (16)	0.0037 (13)	0.0040 (13)	0.0000 (12)
C9	0.0429 (19)	0.0389 (16)	0.0438 (17)	0.0101 (14)	0.0076 (14)	−0.0011 (14)
C7	0.0413 (19)	0.0462 (17)	0.0408 (17)	0.0070 (15)	0.0021 (14)	0.0022 (14)
C6	0.0366 (18)	0.0449 (17)	0.0487 (19)	0.0088 (14)	0.0050 (15)	0.0017 (14)
C8	0.0416 (19)	0.0366 (15)	0.0423 (17)	0.0032 (13)	0.0071 (14)	−0.0019 (13)
C11	0.047 (2)	0.0396 (16)	0.0391 (17)	0.0048 (14)	0.0068 (14)	0.0006 (13)
C10	0.046 (2)	0.0373 (16)	0.0417 (17)	0.0057 (14)	0.0038 (14)	−0.0026 (13)
C16	0.077 (3)	0.0429 (19)	0.049 (2)	−0.0025 (18)	0.011 (2)	0.0005 (15)
C13	0.102 (4)	0.058 (2)	0.046 (2)	−0.002 (2)	0.002 (2)	−0.0045 (18)
C15	0.100 (4)	0.050 (2)	0.055 (2)	0.001 (2)	0.023 (2)	0.0097 (18)
C12	0.072 (3)	0.0444 (19)	0.048 (2)	0.0001 (18)	0.0008 (18)	−0.0010 (16)
C14	0.090 (3)	0.062 (2)	0.045 (2)	0.009 (2)	0.012 (2)	0.0082 (18)
C1	0.079 (3)	0.089 (3)	0.046 (2)	0.021 (3)	0.000 (2)	0.004 (2)

### 5-Benzoyl-2-[(methoxycarbonyl)amino]-1H-1,3-benzodiazol-3-ium dihydrogen phosphate–phosphoric acid (1/1) Geometric parameters (Å, º)

**Table:**

P2—O5	1.580 (2)	C5—C4	1.395 (4)
P2—O6	1.495 (2)	C5—C6	1.379 (4)
P2—O4	1.506 (2)	C4—C9	1.366 (4)
P2—O7	1.548 (2)	C9—H9	0.950 (19)
P1—O10	1.491 (2)	C9—C8	1.389 (4)
P1—O9	1.541 (2)	C7—H7	0.968 (18)
P1—O8	1.531 (2)	C7—C6	1.386 (5)
P1—O11	1.539 (2)	C7—C8	1.417 (4)
O5—H5	0.843 (18)	C6—H6	0.956 (19)
O9—H9A	0.854 (19)	C8—C10	1.487 (4)
O8—H8	0.869 (19)	C11—C10	1.486 (4)
O11—H11	0.842 (19)	C11—C16	1.393 (5)
O7—H7A	0.851 (19)	C11—C12	1.391 (5)
O2—C2	1.197 (4)	C16—H16	0.966 (19)
O1—C2	1.329 (4)	C16—C15	1.384 (5)
O1—C1	1.448 (5)	C13—H13	0.966 (19)
O3—C10	1.236 (4)	C13—C12	1.376 (5)
N2—H2	0.899 (18)	C13—C14	1.379 (6)
N2—C3	1.339 (4)	C15—H15	0.965 (19)
N2—C4	1.399 (4)	C15—C14	1.371 (6)
N3—H3	0.897 (19)	C12—H12	0.952 (19)
N3—C3	1.333 (4)	C14—H14	0.964 (19)
N3—C5	1.397 (4)	C1—H1A	0.98 (2)
N1—H1	0.879 (19)	C1—H1B	0.98 (2)
N1—C2	1.377 (4)	C1—H1C	0.98 (2)
N1—C3	1.357 (4)		
			
O6—P2—O5	110.91 (12)	C4—C9—H9	127 (2)
O6—P2—O4	114.70 (13)	C4—C9—C8	118.0 (3)
O6—P2—O7	106.86 (13)	C8—C9—H9	115 (2)
O4—P2—O5	104.97 (12)	C6—C7—H7	117 (2)
O4—P2—O7	112.45 (14)	C6—C7—C8	121.4 (3)
O7—P2—O5	106.72 (12)	C8—C7—H7	121 (2)
O10—P1—O9	113.59 (13)	C5—C6—C7	117.4 (3)
O10—P1—O8	108.71 (13)	C5—C6—H6	123 (2)
O10—P1—O11	110.19 (14)	C7—C6—H6	119 (2)
O8—P1—O9	106.93 (14)	C9—C8—C7	120.0 (3)
O8—P1—O11	108.89 (14)	C9—C8—C10	117.6 (3)
O11—P1—O9	108.40 (13)	C7—C8—C10	122.4 (3)
P2—O5—H5	116 (3)	C16—C11—C10	121.7 (3)
P1—O9—H9A	115 (3)	C12—C11—C10	119.1 (3)
P1—O8—H8	121 (3)	C12—C11—C16	119.0 (3)
P1—O11—H11	118 (3)	O3—C10—C8	119.2 (3)
P2—O7—H7A	118 (3)	O3—C10—C11	119.7 (3)
C2—O1—C1	114.4 (3)	C11—C10—C8	121.1 (3)
C3—N2—H2	122 (2)	C11—C16—H16	119 (2)
C3—N2—C4	107.5 (3)	C15—C16—C11	119.7 (4)
C4—N2—H2	130 (2)	C15—C16—H16	120 (2)
C3—N3—H3	124 (2)	C12—C13—H13	118 (3)
C3—N3—C5	108.3 (3)	C12—C13—C14	120.3 (4)
C5—N3—H3	128 (2)	C14—C13—H13	121 (3)
C2—N1—H1	121 (2)	C16—C15—H15	120 (3)
C3—N1—H1	117 (2)	C14—C15—C16	120.7 (4)
C3—N1—C2	122.4 (3)	C14—C15—H15	119 (3)
O2—C2—O1	126.3 (3)	C11—C12—H12	117 (2)
O2—C2—N1	124.4 (3)	C13—C12—C11	120.4 (3)
O1—C2—N1	109.3 (3)	C13—C12—H12	123 (2)
N2—C3—N1	125.6 (3)	C13—C14—H14	120 (3)
N3—C3—N2	110.8 (3)	C15—C14—C13	119.8 (4)
N3—C3—N1	123.6 (3)	C15—C14—H14	120 (3)
C4—C5—N3	106.4 (3)	O1—C1—H1A	111 (3)
C6—C5—N3	132.5 (3)	O1—C1—H1B	108 (3)
C6—C5—C4	121.1 (3)	O1—C1—H1C	104 (3)
C5—C4—N2	107.1 (3)	H1A—C1—H1B	114 (5)
C9—C4—N2	130.9 (3)	H1A—C1—H1C	107 (5)
C9—C4—C5	122.1 (3)	H1B—C1—H1C	113 (4)
			
N2—C4—C9—C8	177.9 (3)	C7—C8—C10—O3	156.5 (3)
N3—C5—C4—N2	1.2 (3)	C7—C8—C10—C11	−23.3 (5)
N3—C5—C4—C9	−179.7 (3)	C6—C5—C4—N2	−178.1 (3)
N3—C5—C6—C7	−179.0 (3)	C6—C5—C4—C9	1.0 (5)
C2—N1—C3—N2	−5.9 (5)	C6—C7—C8—C9	1.1 (5)
C2—N1—C3—N3	173.8 (3)	C6—C7—C8—C10	−177.8 (3)
C3—N2—C4—C5	−1.1 (3)	C8—C7—C6—C5	−1.1 (5)
C3—N2—C4—C9	180.0 (3)	C11—C16—C15—C14	−2.2 (8)
C3—N3—C5—C4	−0.9 (4)	C10—C11—C16—C15	−176.3 (4)
C3—N3—C5—C6	178.3 (4)	C10—C11—C12—C13	178.9 (4)
C3—N1—C2—O2	−2.0 (5)	C16—C11—C10—O3	141.1 (4)
C3—N1—C2—O1	177.9 (3)	C16—C11—C10—C8	−39.1 (5)
C5—N3—C3—N2	0.3 (4)	C16—C11—C12—C13	3.0 (7)
C5—N3—C3—N1	−179.5 (3)	C16—C15—C14—C13	2.5 (8)
C5—C4—C9—C8	−0.9 (5)	C12—C11—C10—O3	−34.6 (5)
C4—N2—C3—N3	0.5 (4)	C12—C11—C10—C8	145.2 (4)
C4—N2—C3—N1	−179.7 (3)	C12—C11—C16—C15	−0.6 (6)
C4—C5—C6—C7	0.1 (5)	C12—C13—C14—C15	0.0 (8)
C4—C9—C8—C7	−0.1 (5)	C14—C13—C12—C11	−2.8 (8)
C4—C9—C8—C10	178.9 (3)	C1—O1—C2—O2	−4.3 (6)
C9—C8—C10—O3	−22.4 (5)	C1—O1—C2—N1	175.9 (3)
C9—C8—C10—C11	157.8 (3)		

### 5-Benzoyl-2-[(methoxycarbonyl)amino]-1H-1,3-benzodiazol-3-ium dihydrogen phosphate–phosphoric acid (1/1) Hydrogen-bond geometry (Å, º)

**Table:**

*D*—H···*A*	*D*—H	H···*A*	*D*···*A*	*D*—H···*A*
O5—H5···O10^i^	0.84 (2)	1.74 (2)	2.578 (3)	175 (4)
O9—H9*A*···O6^ii^	0.85 (2)	1.72 (2)	2.568 (3)	177 (5)
O8—H8···O4	0.87 (2)	1.68 (2)	2.544 (3)	171 (4)
O11—H11···O10^iii^	0.84 (2)	1.79 (2)	2.629 (3)	178 (4)
O7—H7*A*···O6^iii^	0.85 (2)	1.79 (2)	2.594 (3)	157 (4)
N2—H2···O2	0.90 (2)	2.10 (3)	2.680 (4)	122 (3)
N3—H3···O4^iv^	0.90 (2)	1.82 (2)	2.717 (3)	177 (3)
N1—H1···O5^iv^	0.88 (2)	1.96 (2)	2.831 (3)	174 (3)

Symmetry codes: (i) *x*−1, −*y*+1/2, *z*−1/2; (ii) *x*, −*y*+1/2, *z*+1/2; (iii) *x*−1, *y*, *z*; (iv) *x*+1, *y*, *z*.
