# Supplementary material for: Supra­molecular assembly of mebendazolium and di­hydrogen phosphate ions in a new anthelmintic salt
Source: Acta Crystallogr E Crystallogr Commun. 2025 Feb 4;81(Pt 3):195–9. doi: 10.1107/S2056989025000714 (PMC11891589; doi:10.1107/S2056989025000714)
Supplement: Supplementary file 3 [file e-81-00195-sup4.pdf]

Supramolecular assembly of mebendazolium and dihydrogenphosphate ions in a new anthelmintic salt

Eduardo L. Gutierrez,[a]* Marcos Russo,[b] Griselda E. Narda,[b] Elena V. Brusau,[b] Alejandro P. Ayala[c] and Javier Ellena[d]

[a]Instituto de Investigaciones en Tecnología Química (INTEQUI), CONICET – Área de Química Orgánica, Facultad de Química, Bioquímica y Farmacia, Universidad Nacional de San Luis, D5700APC, San Luis, Argentina, [b]Instituto de Investigaciones en Tecnología Química (INTEQUI), CONICET – Área de Química General e Inorgánica, Facultad de Química, Bioquímica y Farmacia, Universidad Nacional de San Luis, D5700APC, San Luis, Argentina, [c]Departamento de Física, Universidade Federal do Ceará, 60.440-900, Fortaleza, CE, Brasil, and [d]Instituto de Física de São Carlos, Universidade de São Paulo, 13.566-590, São Carlos, SP, Brasil.

*Correspondence e-mail: egutierrez@unsl.edu.ar

Supplementary Information

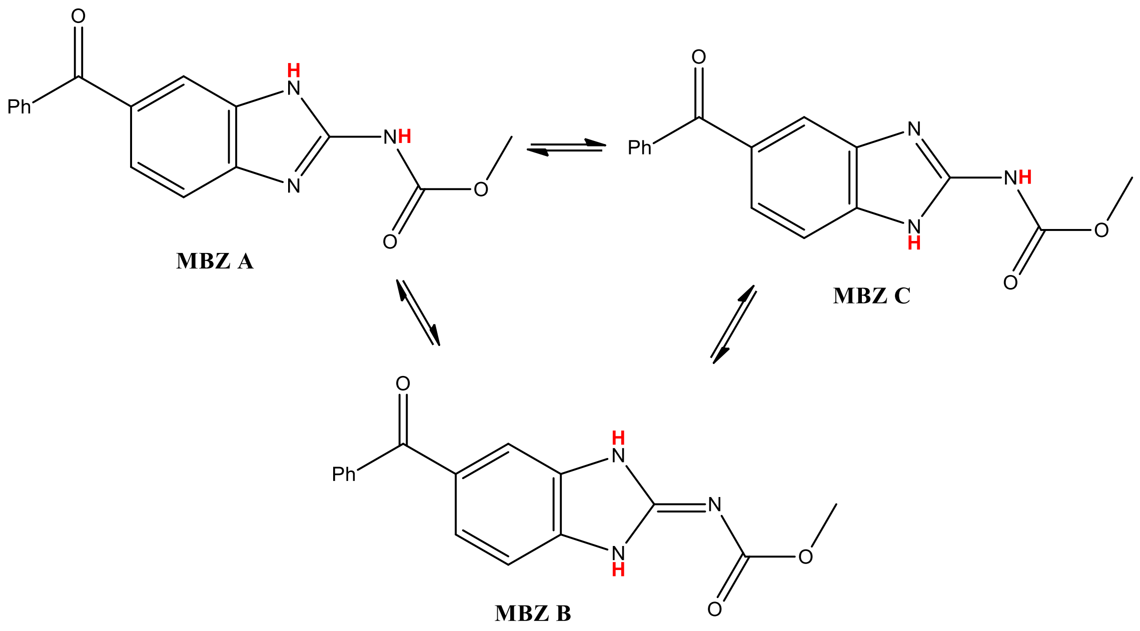

Scheme S1. Tautomeric equilibria between the three tautomers found in MBZ A, MBZ B, and MBZ C desmotropes respectively

Bond length analysis

Bond length changes in the imidazole ring and the carbamate moiety confirmed that protonation of the API indeed occurs as expected (Scheme S2). In the unprotonated form of MBZ, as found in form C (Martins, 2009), both bonds C3–N2 (1.304(11) Å) and C3–N3 (1.349(10) Å) are significantly different. The bond C3–N2 exhibits a higher double bond character, as shown in the canonical structure in Scheme S2. However, in the mebendazolium cation present in $MBZH·PO_2(OH)_2·PO(OH)_3$, resonance effects are more important since protonation on the molecule leads to the decreasing in the bond length difference being equals within experimental error [C3–N2 (1.333(4) Å) and C3–N3 (1.339(4) Å)]. Further evidence of the protonation of the API is given by the bond length C3–N1. While this bond is 1.372(11) Å in the neutral molecule, it is shortened in $MBZH^+$ (1.357(4) Å) due to a displacement of the electron density from N1 towards the positively charged ring.

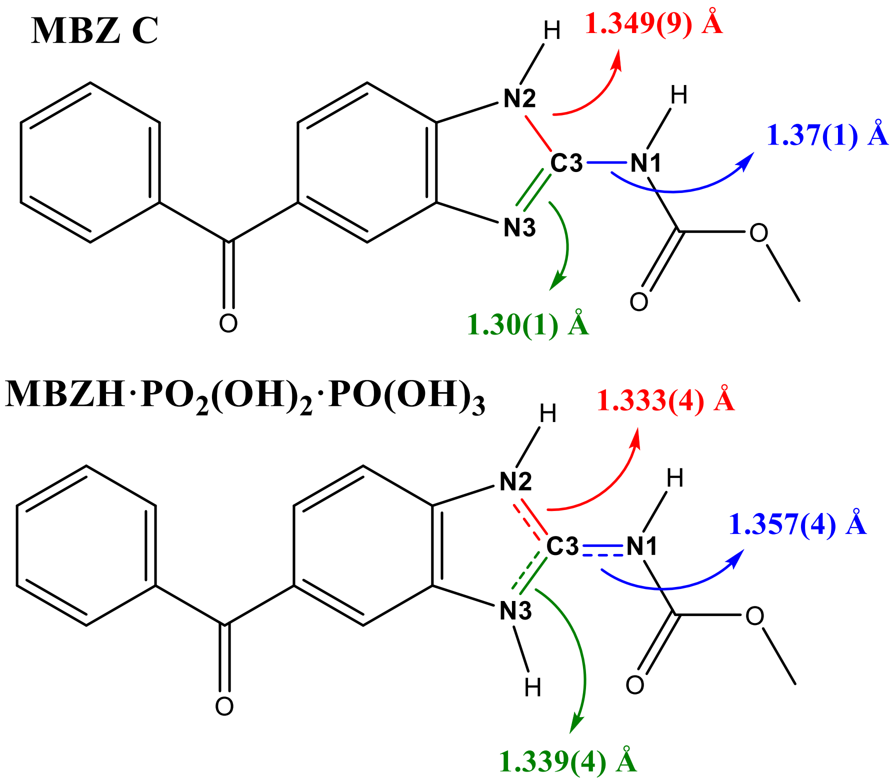

Scheme S2. Effect of protonation on bond lengths within the MBZ molecule/ion.

ΔpK_a calculation

ΔpK_a was calculated on the basis of pK_a values of mebendazolium cation (3.43) (Wan, 2003) and phosphoric acid first ionization constant (2.12) (Lopez, 2001):

$$\Delta \mathrm{p}K_a \equiv \mathrm{p}K_a(protonated\ base) - \mathrm{p}K_a(acid)$$

$$\Delta \mathrm{p}K_a = 3.43 - 2.12 = 1.31$$

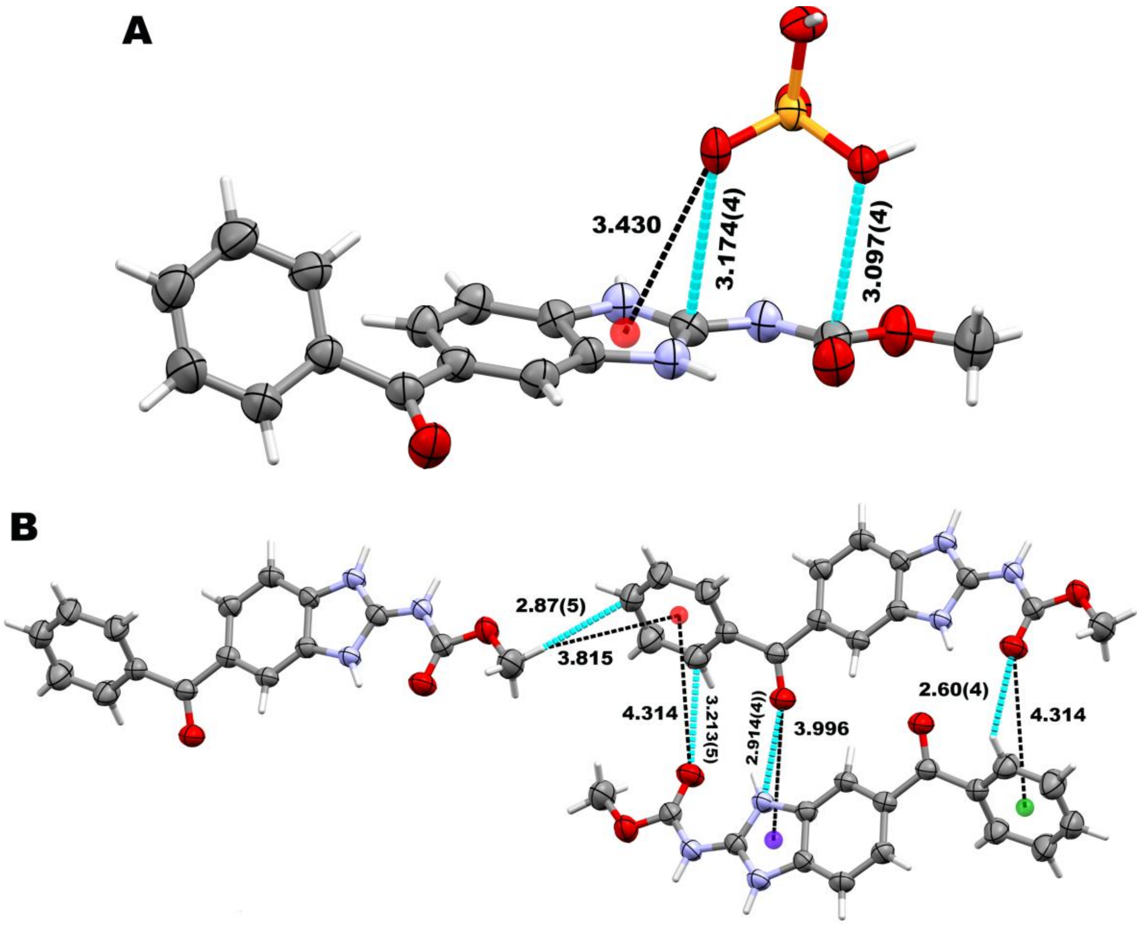

Figure S1A. Interactions between a MBZH$^+$ cation and a $PO_2(OH)_2^-$ anion approx. along the *b* axis. **B.** C–H···π and carbonyl···π interactions relating adjacent MBZH$^+$ cations. Distances are give in Å.

Table S1

Hydrogen-bonding features of the $R_2^2(8)$ supramolecular motif found in mebendazolium salts

$MBZH^+$ salts	HB	**D–H** / Å	**H···A** / Å	**D···A** / Å	**D–H···A** / °
Dihydrogenphosphate	A	0.88(3)	1.95(3)	2.831(4)	174(3)
(This work)	B	0.90(3)	1.82(3)	2.717(4)	176(3)
Mesylate	A	0.860	1.870	2.715(8)	167.0
(DOFBEJ)	B	0.860	1.961	2.785(7)	160.2
perchlorate	A	0.860	2.043	2.875(2)	162.6
(RACFOU)	B	0.860	1.943	2.787(5)	166.8
trifluoroacetate	A	0.880	1.796	2.672(2)	173.3
(PIDKOE)	B	0.880	1.761	2.650(2)	165.0
Formiate*	A	0.879	1.901	2.779(5)	176.6
(PIDKIY)	B	0.880	1.840	2.704(5)	166.7
methylsulphate	A	0.860	1.901	2.736(3)	163.3
(RACFUA)	B	0.860	1.891	2.742(3)	169.7
nitrate	A	0.860	2.081	2.759(3)	135.3
(RERNIO)	B	0.860	1.867	2.720(2)	171.6
maleate*	A	1.06(5)	1.71(5)	2.757(4)	173(4)
(VEVPUJ)	B	0.91(4)	1.72(4)	2.627(4)	175(4)
methyloxalate	A	0.79(4)	2.01(4)	2.798(4)	170(4)
(VEVQAQ)	B	1.06(4)	1.50(4)	2.559(3)	174(3)

*These compounds present more than one distinct $R_2^2(8)$ supramolecular motif with slightly differences in the hydrogen bonds features. One of them are selected for this table as an example for each case.

HB: hydrogen-bond. D: donor. A: acceptor.

45

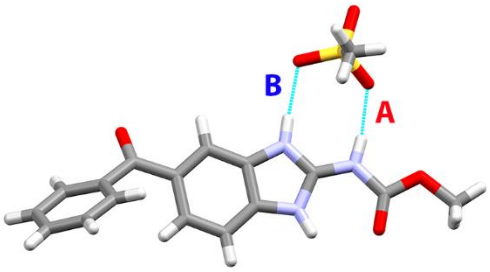

Mebendazolium mesylate (DOFBEJ)
(Gutiérrez, 2024)

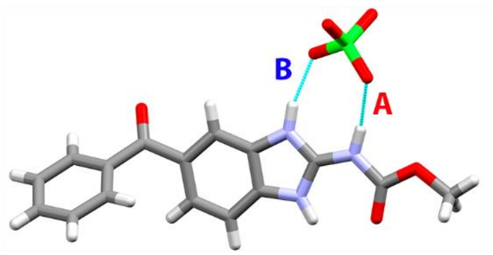

Mebendazolium perchlorate (RACFOU)
(Gutiérrez, 2020)

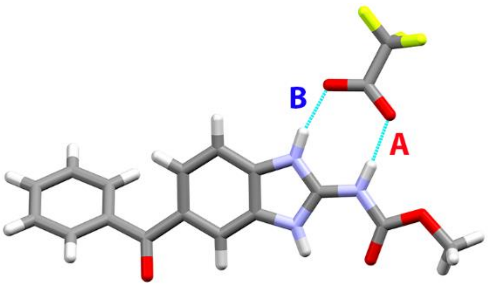

Mebendazolium trifluoroacetate (PIDKOE)
(Chen, 2013)

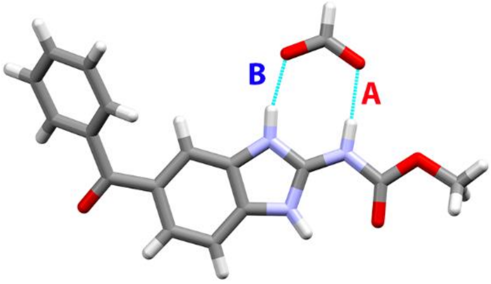

Mebendazolium formiate (PIDKIY)
(Chen, 2013)

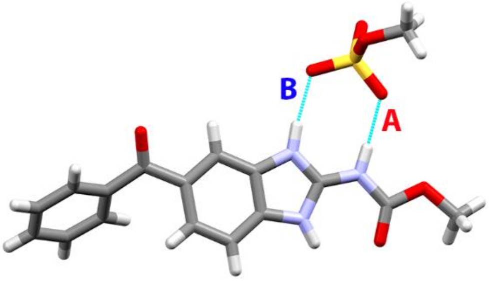

Mebendazolium methylsulphate (RACFUA)
(Gutiérrez, 2020)

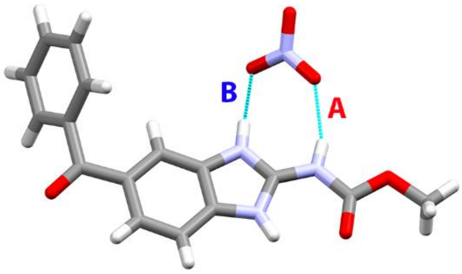

Mebendazolium nitrate (RERNIO)
(Gutiérrez, 2018)

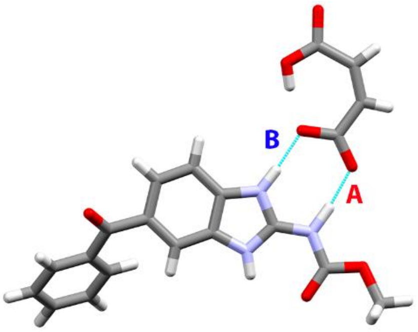

Mebendazolium maleate (VEVPUJ)
(Chen, 2012)

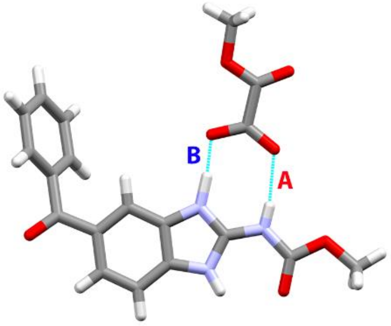

Mebendazolium methyloxalate (VEVQAQ)
(Chen, 2012)

Figure S2. $R_2^2(8)$ supramolecular motifs found in mebendazolium salts
(Color code: C: grey, H: white, N: purple, O: red, S: yellow, F, green.)

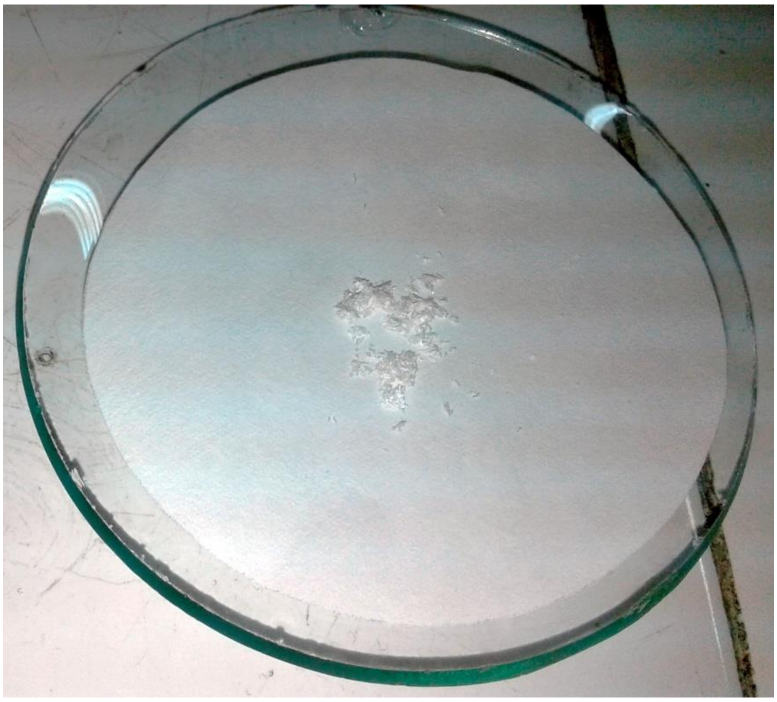

Figure S3. $MBZH\cdot PO_2(OH)_2\cdot PO(OH)_3$ crystals

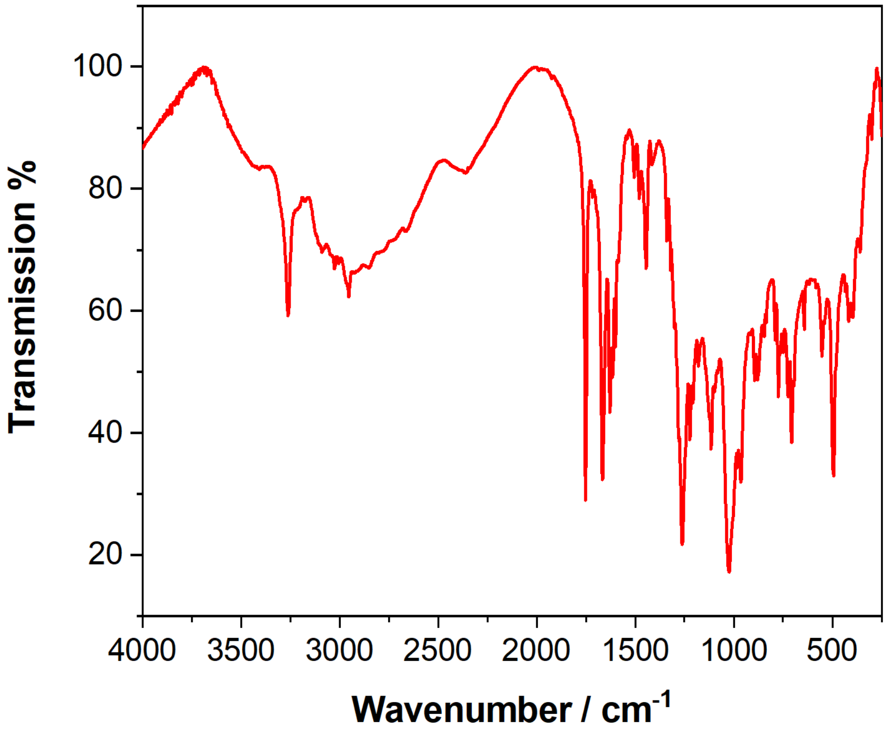

Figure S4. FT-IR spectra of MBZH·$PO_2(OH)_2$·$PO(OH)_3$

Table S2
Assignment of selected vibrational modes of the FTIR spectrum

Modes	Wavenumbers / cm^{-1}
ν (N–H) (carbamate)	3261
$ν_{as}$ (C–H) (aromatic)	3093
$ν_s$ (C–H) (aromatic)	3026
$ν_{as}$ (C–H) (aliphatic)	2953
$ν_s$ (C–H) (aliphatic)	2852
ν (C=O) (carbamate)	1753
ν (P–OH)	1200–900 (several bands)
$ν_{as}$ (O–P–O)	553<br>496

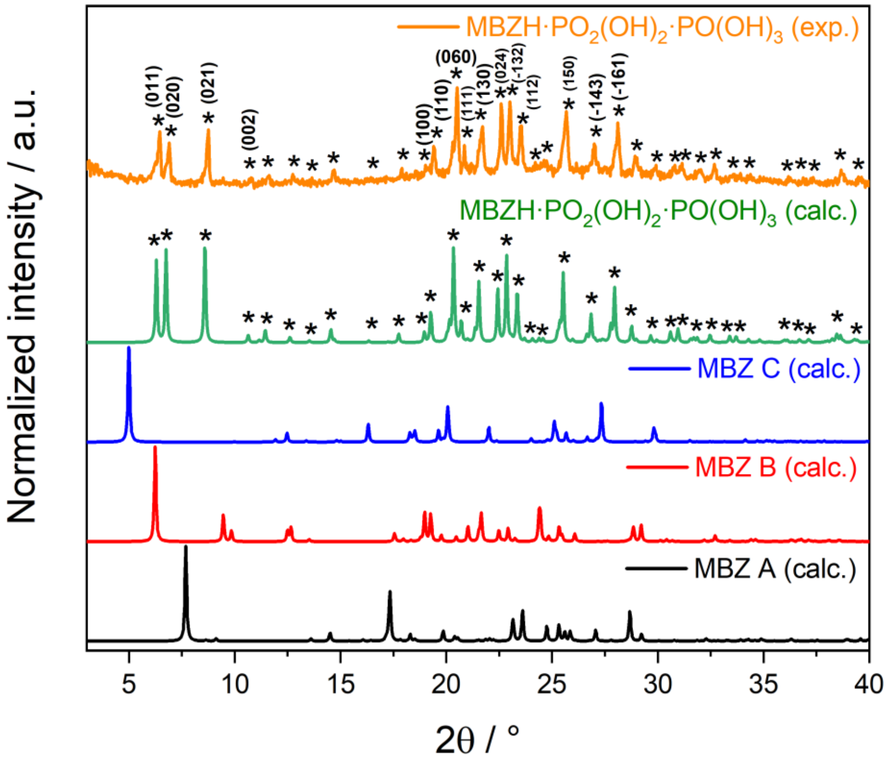

Figure S5. Experimental powder X-ray diffraction pattern of $MBZH \cdot PO_2(OH)_2 \cdot PO(OH)_3$, compared with those calculated from single-crystal XRD data

Table S3

Miller indexes and position of the main reflections on the experimental PXRD pattern

h	k	l	2θ / ° (obs.)
0	1	1	6.45
0	2	0	6.90
0	2	1	8.76
0	0	2	10.80
1	0	0	19.02
1	1	0	19.41
0	6	0	20.52
1	1	1	20.85
1	3	0	21.72
0	2	4	22.59
$\bar{1}$	3	2	23.01
1	1	2	23.55
1	5	0	25.68
$\bar{1}$	4	3	27.00
$\bar{1}$	6	1	28.11

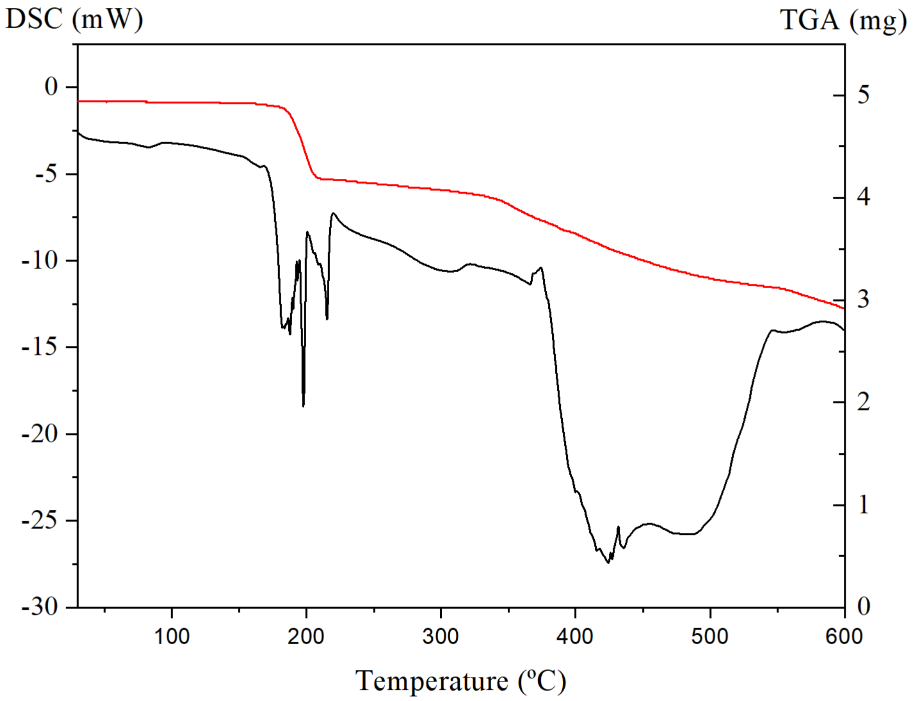

Figure S6. TGA and DSC curves of MBZH·$PO_2(OH)_2$·PO(OH)

Bibliography

Chen, J.-M., Wang, Z.-Z. & Lu, T.-B. (2012). *CrystEngComm*, **14**, 6221–6229.

Chen, J. & Lu., T. (2013) & *Chin. J. Chem. Phys.*, **31**(5), 635–640.

Gutiérrez, E. L., Souza, M. S., Diniz, L. F. & Ellena, J. (2018). *J. Mol. Struct.* **1161**, 113–121.

Gutiérrez, E. L., Godoy, A. A., Narda, G. E. & Ellena, J. (2020). *CrystEngComm.* **22**, 6559–6568.

Gutiérrez, E. L., Godoy, A. A., Brusau, E. V., Vega, D., Narda, G. E., Suárez, S. & Di Salvo, F. (2024). *RSC Adv.* **14**, 181–192.

Lopez, X., Schaefer, M., Dejaegere, A. & Karplus, M. (2002). *J. Am. Chem. Soc.*, **124**, 5010–5018

Martins, F. T., Neves, P. P., Ellena, J., Camí, G. E., Brusau, E. V. & Narda, G. E. (2009). *J. Pharm. Sci.* **98**(7), 2336c2344.

Wan, H., Holmén, A. G., Wang, Y., Lindberg, W., Englund, M., Någård, M. B. & Thompson, R. A. (2003). *Rapid Commun. Mass Spectrom.*, **17**, 2639–2648.
